# GPSFun: geometry-aware protein sequence function predictions with language models

**DOI:** 10.1093/nar/gkae381

**Published:** 2024-05-13

**Authors:** Qianmu Yuan, Chong Tian, Yidong Song, Peihua Ou, Mingming Zhu, Huiying Zhao, Yuedong Yang

**Affiliations:** School of Computer Science and Engineering, Sun Yat-sen University, Guangzhou, Guangdong 510000, China; School of Computer Science and Engineering, Sun Yat-sen University, Guangzhou, Guangdong 510000, China; School of Computer Science and Engineering, Sun Yat-sen University, Guangzhou, Guangdong 510000, China; School of Computer Science and Engineering, Sun Yat-sen University, Guangzhou, Guangdong 510000, China; School of Computer Science and Engineering, Sun Yat-sen University, Guangzhou, Guangdong 510000, China; Sun Yat-sen Memorial Hospital, Sun Yat-sen University, Guangzhou, Guangdong 510000, China; School of Computer Science and Engineering, Sun Yat-sen University, Guangzhou, Guangdong 510000, China

## Abstract

Knowledge of protein function is essential for elucidating disease mechanisms and discovering new drug targets. However, there is a widening gap between the exponential growth of protein sequences and their limited function annotations. In our prior studies, we have developed a series of methods including GraphPPIS, GraphSite, LMetalSite and SPROF-GO for protein function annotations at residue or protein level. To further enhance their applicability and performance, we now present GPSFun, a versatile web server for Geometry-aware Protein Sequence Function annotations, which equips our previous tools with language models and geometric deep learning. Specifically, GPSFun employs large language models to efficiently predict 3D conformations of the input protein sequences and extract informative sequence embeddings. Subsequently, geometric graph neural networks are utilized to capture the sequence and structure patterns in the protein graphs, facilitating various downstream predictions including protein–ligand binding sites, gene ontologies, subcellular locations and protein solubility. Notably, GPSFun achieves superior performance to state-of-the-art methods across diverse tasks without requiring multiple sequence alignments or experimental protein structures. GPSFun is freely available to all users at https://bio-web1.nscc-gz.cn/app/GPSFun with user-friendly interfaces and rich visualizations.

## Graphical Abstract

**Graphical Abstract:**
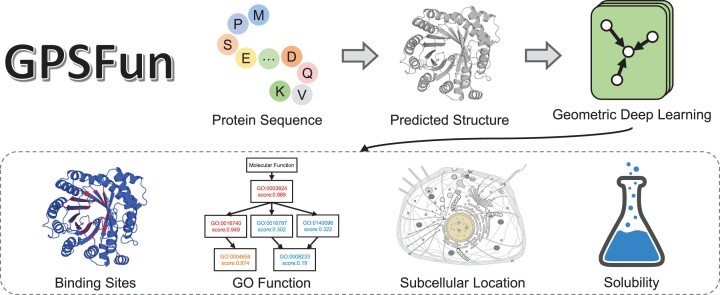

## Introduction

Knowledge of protein function is crucial for comprehending metagenome functions, unraveling disease mechanisms and discovering new drug targets (1). Since biochemical experiments for protein function determination are expensive, time-consuming, and of low throughput (2), there is currently a widening gap between the rapid expansion of protein sequences and their limited function annotations (3). To this end, numerous computational tools have been developed for protein function predictions at residue and protein levels, such as protein–ligand binding sites (4–9), gene ontologies (GO) (10–14), subcellular locations (15–17) and protein solubility (18–20).

Despite the abundance of protein function predictors designed for various tasks, a one-stop comprehensive platform that offers high-quality predictions covering a wide range of functions is lacking. Furthermore, many existing sequence-based methods, such as TargetS (21), heavily rely on multiple sequence alignments (MSA), which are computationally expensive and futile for orphan proteins that lack close homologs. While our previous studies, LMetalSite (9) and SPROF-GO (12), have overcome this issue by substituting MSA with language model representations, the absence of structural information still presents an opportunity for enhancing accuracy. By comparison, experimental structure-based approaches encoding protein structures via graph neural networks (GNN) (4–7,22–25) are often more effective. Nevertheless, most of these methods have not yet fully explored the geometry within the structure. More importantly, structure-based methods are not applicable to novel proteins with unsolved structures. Although our previously developed GraphSite (8) has shown the feasibility of leveraging AlphaFold2-predicted structures (26) for DNA-binding site prediction, the computationally intensive structure prediction pipeline hinders its application to sequences absent from the AlphaFold Protein Structure Database (27).

Based on the recent prosperity of protein language models (28,29), ESMFold (30) has emerged as a promising alternative to AlphaFold2, which replaces MSA with a large-scale pre-trained protein language model to significantly accelerate the prediction speed while maintaining comparable accuracy. To facilitate protein structure modeling, geometric deep learning has recently flourished in protein structure pre-training (31), protein design (32,33), protein docking (34,35), and binding site prediction (4–6,22). Building upon these recent advancements, it is promising to further enhance the applicability and performance of our previously well-validated methods for protein function annotations (7–9,12,18).

Here, we present GPSFun, a versatile web server for Geometry-aware Protein Sequence Function annotations, including protein binding sites for various ligands (i.e. DNA, RNA, peptide, protein, ATP, HEM, Zn^2+^, Ca^2+^, Mg^2+^ and Mn^2+^), gene ontologies, subcellular locations and protein solubility. Specifically, starting from the protein sequences in FASTA format, GPSFun employs pre-trained language models to efficiently predict the 3D conformations of the proteins and extract informative sequence embeddings. Subsequently, geometric GNNs are utilized to synergistically capture the sequence and structure patterns in the protein graphs for diverse downstream tasks. Notably, GPSFun is independent of MSA and experimental protein structures, enabling fast and accurate predictions from sequences. Experiments demonstrate that GPSFun substantially outperforms state-of-the-art methods across various tasks. By providing user-friendly interfaces and rich interactive visualizations, GPSFun serves as a reliable and efficient tool for biologists and chemists. The GPSFun web server is freely available to all users at https://bio-web1.nscc-gz.cn/app/GPSFun.

## Materials and methods

### Benchmark datasets

The benchmark datasets for assessing binding site predictions of DNA, RNA, peptide, ATP and HEM are compiled from BioLiP (36). For each ligand, we collected the corresponding binding proteins with resolutions ≤3.0 Å and lengths ranging from 50 to 1500 released on 29 March 2023. We combined the binding site annotations of identical sequences and then removed redundant sequences sharing identity >25% over 30% alignment coverage using CD-HIT (37). Subsequently, each benchmark dataset was split into a training set with proteins released before 1 January 2021, and an independent test set with proteins released between 1 January 2021 and 29 March 2023. The datasets of protein-protein and protein-metal-ion (Zn^2+^, Ca^2+^, Mg^2+^ and Mn^2+^) binding sites are directly obtained from our previous studies (7,9). To evaluate GO, subcellular localization, and solubility predictions, we adopted the datasets from (11), (17) and (20), respectively. More details of these benchmark datasets are provided in Supplementary Note S1 and Supplementary Tables S1–S4.

### The workflow of GPSFun

**Figure 1.**
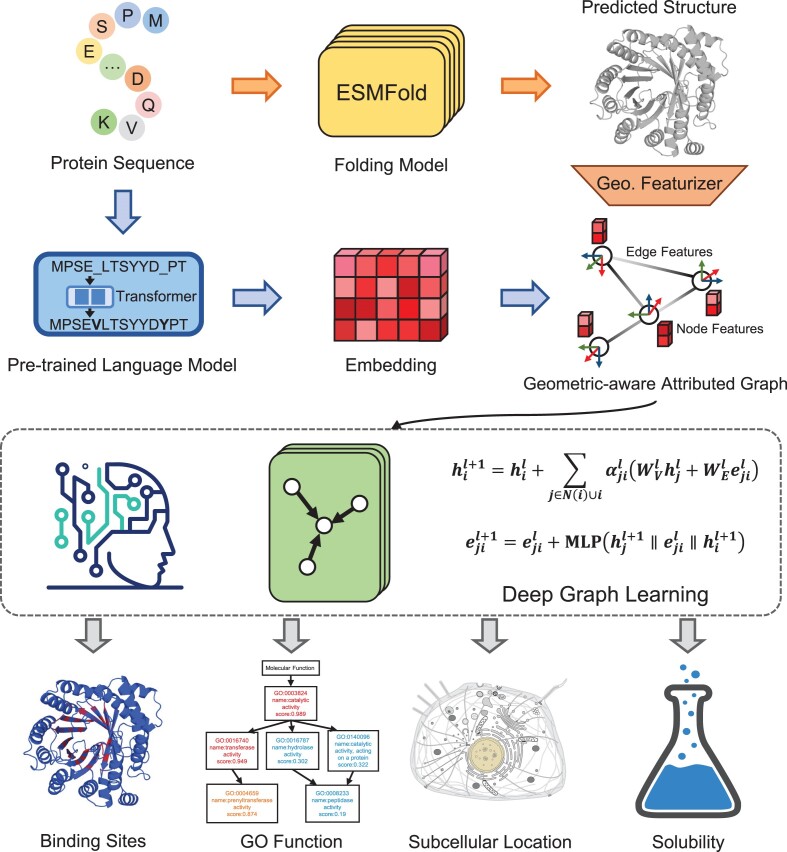
The workflow of GPSFun. For an input sequence, GPSFun first adopts the language model-based folding algorithm ESMFold to efficiently predict the 3D conformation of the protein. Then, another pre-trained protein language model is used to extract informative sequence embedding, and a geometric featurizer is employed to capture the residual and relational geometric contexts in the predicted structure. The resulting geometric-aware protein attributed graph is fed into a set of deep graph neural networks to discover high-level patterns for various downstream tasks, including protein–ligand binding site, GO function, subcellular localization and solubility predictions.

The workflow of GPSFun is shown in Figure 1. For an input sequence, GPSFun first adopts the language model-based folding algorithm ESMFold (30) to predict the 3D conformation of the protein. Then, another pre-trained protein language model ProtTrans (version: ProtT5-XL-U50) (29) is used to extract sequence embedding, which is further normalized via min-max normalization as in (9,12). Subsequently, a geometric featurizer is employed to capture the residual and relational geometric contexts in the predicted structure. We also calculate the relative solvent accessibility and secondary structure profile from the predicted structure using DSSP (38) as done in our previous works (7,8). The resulting geometric-aware protein attributed graph is input to a set of GNNs to discover high-level patterns for various downstream tasks, including protein–ligand binding site, GO function, subcellular localization and solubility predictions.

#### The geometric featurizer

GPSFun represents a protein as a radius graph where residues constitute the nodes and adjacent nodes (distance between C_α_ < 15 Å) are connected by edges. An end-to-end featurizer is utilized to extract geometric features similar to (33), except that we additionally encode the sidechain conformations of the residues. Specifically, a local coordinate system is first defined at each residue based on the relative positions of the backbone C_α_, N and C atoms. Then, several SE(3)-invariant geometric features are derived to capture the arrangements of backbone and sidechain atoms in or between residues. The geometric node features consist of intra-residue distances between any two atoms, relative directions of other inner atoms to C_α_, as well as bond and torsion angles. The geometric edge features consist of inter-residue distances between any two atoms from the adjacent residues respectively, relative directions of all atoms in the neighboring residue to C_α_ of the central residue, as well as rotation angles between the two reference frames of the neighboring nodes. To encode the sidechain conformations, the centroids of the heavy sidechain atoms are calculated, which participate in the above feature calculations as regular atoms. The detailed definitions of the geometric features are given in Supplementary Note S2.

#### The deep graph neural networks

Given a protein attributed graph containing ProtTrans, DSSP and geometric node features, as well as geometric edge features, several GNN layers are adopted to learn the high-level residue representations. Specifically, we denote the hidden feature vectors of node $i$ and edge $j \to i$ in layer $l$ as $h_i^l$ and $e_{ji}^l$, respectively. To update node $i$, the message passing in layer $l$ is performed as follows:


(1) \begin{eqnarray*}\hat{h}_i^{l + 1} = h_i^l + \mathop \sum \limits_{j \in N\left( i \right)\, \cup\, i} \alpha _{ji}^l\left( {W_V^lh_j^l + W_E^le_{ji}^l} \right)\end{eqnarray*}

where the attention coefficient $\alpha _{ji}^l$ from node $j$ to $i$ is calculated by:


(2) \begin{eqnarray*}\left\{ {\begin{array}{@{}*{1}{c}@{}} {w_{ji}^l = \frac{{{{\left( {W_Q^lh_i^l} \right)}}^T\left( {W_K^lh_j^l + W_E^le_{ji}^l} \right)}}{{\sqrt d }}}\\ {\alpha _{ji}^l = \frac{{\exp w_{ji}^l}}{{\mathop \sum \nolimits_{k \in N\left( i \right)\,\cup\,i} \exp w_{ki}^l}}} \end{array}} \right.\end{eqnarray*}

$W_Q^l$ , $W_K^l$, $W_V^l$ and $W_E^l$ are learnable weight matrices, and $N( i )$ denotes the neighbours of node $i$. Then we update the features of an edge using its connecting nodes:


(3) \begin{eqnarray*}e_{ji}^{l + 1} = e_{ji}^l + {\mathrm{MLP}}\left( {\hat{h}_j^{l + 1}\parallel e_{ji}^l\parallel \hat{h}_i^{l + 1}} \right)\end{eqnarray*}

where $\parallel$ denotes vector concatenation and MLP denotes multilayer perceptron. We also exploit the global node update module in (33) to capture the global information.

### Training and evaluation

To train the models for protein–ligand binding site, subcellular localization, and solubility predictions, we conducted five-fold cross-validation on the training sets. For GO prediction, the models were trained on the training sets using five different random seeds and evaluated on the pre-defined validation sets. All hyperparameters were optimized via grid search based on the performance of the validation sets. In the test phase, all five trained models (from cross-validation or different seeds) were used to make predictions, which were averaged as the final prediction of GPSFun. Multi-task learning was employed to train the binding site data for different ligands concurrently as in LMetalSite (9), and we integrated the native and predicted structures to augment the training process. The homology-based label-diffusion in SPROF-GO (12) is also incorporated into GPSFun for GO and subcellular localization predictions. We adopted Pytorch (39) to implement GPSFun, and Adam optimizer (40) for model optimization with binary cross entropy loss. More details of the architecture and training strategy of GPSFun are provided in Supplementary Table S5. Besides, the implementations of the baseline methods are detailed in Supplementary Note S3. Consistent with previous studies, we use recall (Rec), precision (Pre), accuracy (Acc), Jaccard, F1-score (F1), maximum protein-centric F-measure (F_max_), Matthews correlation coefficient (MCC), area under the receiver operating characteristic curve (AUC), and area under the precision-recall curve (AUPR) to evaluate the prediction performance, whose detailed definitions are given in Supplementary Note S4.

### Web server implementation

GPSFun is run on a nginx (https://nginx.org/) server, with a backend based on Go (https://go.dev/) and a frontend based on Vue 3 (https://vuejs.org/). The combination of MySQL (https://www.mysql.com/) and MongoDB (https://www.mongodb.com/) is employed as the database solution. The interactive user interface components are provided by Element Plus (https://element-plus.gitee.io/en-US/). Protein structures are visualized using Mol* (41) (https://molstar.org/), and the GO function predictions are visualized by the directed acyclic graphs (DAG) based on Graphviz (https://graphviz.org/). The users’ submitted jobs are queued and then run on a cluster of NVIDIA Tesla V100 GPUs (16 GB).

## Results

### The GPSFun web server

The GPSFun website (https://bio-web1.nscc-gz.cn/app/GPSFun) is free and open to all users and there is no login requirement. GPSFun neither utilizes cookies nor collects any personal information. GPSFun is compatible with most web browsers including Microsoft Edge, Google Chrome, Apple Safari and Mozilla Firefox across major operating systems including Windows, MacOS and Linux.

#### Inputs

**Figure 2.**
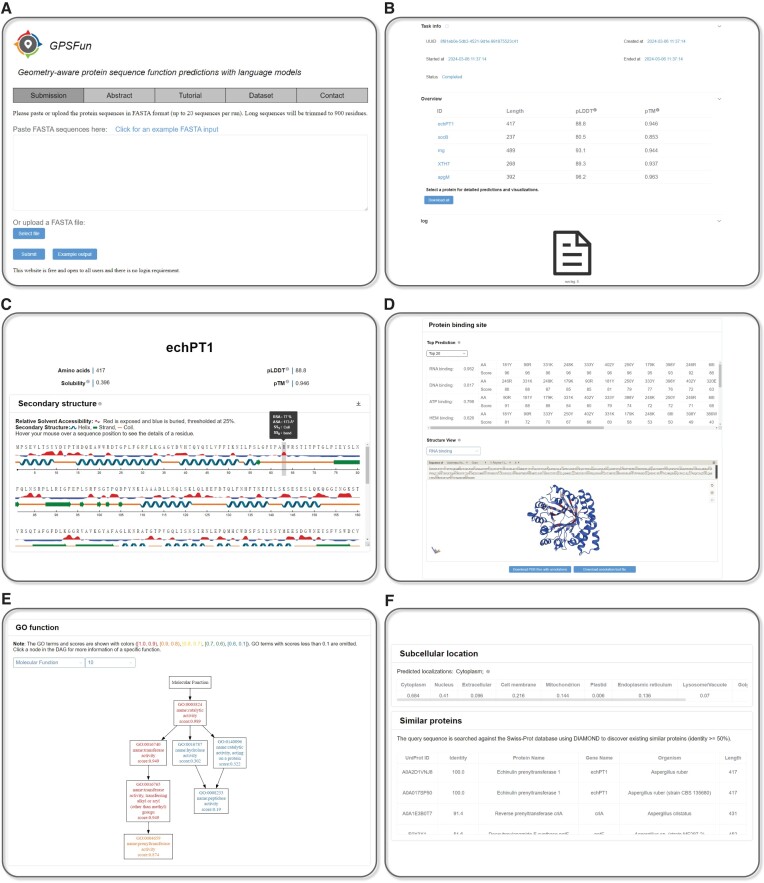
The GPSFun web server. (**A**) The home page of GPSFun. (**B**) The task page of GPSFun with an overview of the submitted proteins and a running log. (C–F) The outputs of GPSFun for the example input (echPT1 gene). (**C**) The confidence metrics and the secondary structure visualizations of the ESMFold-predicted structure. The solubility prediction is also provided. (**D**) Visualizations of the protein–ligand binding site predictions. (**E**) Visualizations of the GO function predictions. (**F**) The subcellular localization predictions, as well as cross-links to other similar proteins in Swiss-Prot.

The home page of GPSFun is shown in Figure 2A, where users can use the navigation bar to submit data, browse the brief introduction of GPSFun, read the detailed tutorial of the server, and download the datasets for training and evaluating GPSFun. To start, users can either paste the protein sequences of interest into the text box or upload a file in FASTA format. Batch predictions are supported for up to 20 proteins. An example input can be automatically loaded with a simple click. After submitting the example input or clicking the ‘Example output’ button, the prediction results for the example sequences will be displayed for demonstration.

#### Outputs

Once the sequences are submitted, users will be directed to a task page similar to Figure 2B. Users can bookmark this page to retrieve their results within 2 months. Typically, the initialization of the environment and loading of all pre-trained models require less than 5 min, while the annotation for a protein with 500 residues takes about 2 min. Upon task completion, a log file is available. Importantly, an overview of the submitted proteins is presented, including protein ID, length, predicted local distance difference test (pLDDT) and predicted TM-score (pTM) estimated by ESMFold. Higher scores of pLDDT and pTM indicate greater confidence in the predicted structures. Users can select a protein for detailed predictions and visualizations, or click the download button below to obtain the predictions for all proteins. Here, we discuss the outputs of GPSFun using the prediction results for the echPT1 gene of Aspergillus ruber (UniProt (42) ID: A0A2D1VNJ8) as an example.

The result page of GPSFun is divided into five sections. It starts with the basic information of the target protein including ID, length, pLDDT, pTM and the predicted solubility by GPSFun (Figure 2C). The secondary structure and relative solvent accessibility calculated from the ESMFold-predicted structure using DSSP are also visualized, where users can further hover the mouse over a sequence position to explore the detailed properties of a residue. The second section (Figure 2D) displays the protein binding site annotations covering ten available ligand types including DNA, RNA, peptide, protein, ATP, HEM, Zn^2+^, Ca^2+^, Mg^2+^ and Mn^2+^. To explore the ligand-binding hotspots, users can examine the top *n* residues with the highest predicted scores in an interactive form. Notably, a structure view panel exhibits the predicted structure along with the GPSFun-predicted ligand-binding propensities. The confidence of the predictions is represented with a gradient of color from blue for non-binding to red for binding. The third section (Figure 2E) illustrates the GO function annotations regarding molecular function (MF), biological process (BP), and cellular component (CC). The hierarchy of the predictions is visualized by a DAG with nodes displayed in different colors based on the predictive scores of the GO terms. Users can click a node in the DAG to explore detailed information on the AmiGO website (https://amigo.geneontology.org/amigo). The fourth section presents the subcellular localization annotations of the protein (Figure 2F). Since similar sequences tend to share similar functions, the last section (Figure 2F) provides cross-links to other similar proteins in Swiss-Prot (42) for reference based on DIAMOND (43).

### Validation

For protein–ligand binding site predictions, we compared GPSFun with state-of-the-art sequence-based methods including GraphSite (8), PepBind (44), PepBCL (45), TargetS (21), and LMetalSite (9), as well as experimental structure-based methods including GraphBind (23), GeoBind (22), aaRNA (46), PepNN (47), MaSIF-site (4), GraphPPIS (7), ScanNet (5), DELIA (48) and IonCom (49). As shown in Table 1 and Supplementary Figures S1 and S2, GPSFun surpasses all competing methods in AUPR by over 17.6%, 14.2%, 55.0%, 1.9%, 29.3%, 12.0%, 6.8%, 17.5%, 16.8% and 15.0% in the independent test sets of DNA, RNA, peptide, protein, ATP, HEM, Zn^2+^, Ca^2+^, Mg^2+^ and Mn^2+^, respectively. To further illustrate the effectiveness of sequence embeddings and predicted structures from language models, we conducted ablation studies as shown in Supplementary Table S6. By employing ProtTrans embeddings as sequence features instead of the MSA profiles we previously used (7,8), an increase of 4.2% in the average AUPR across the ten ligands is obtained. On the other hand, removing the structure information causes a substantial performance drop of 19.3% in the average AUPR. In addition, removal of the geometric featurizer within GPSFun also results in a considerable decline (11.5%) in the average AUPR, underscoring the significance of GPSFun's perception of protein geometry.

**Table 1.** Performance comparison of GPSFun with state-of-the-art methods on the ligand-binding site test sets

Test set	Method	Rec	Pre	Acc	F1	MCC	AUC	AUPR
DNA	GraphBind	0.607	0.355	0.914	0.448	0.422	0.884	0.424
	GeoBind	0.520	0.442	0.935	0.478	0.445	0.896	0.443
	GraphSite	0.493	0.450	0.936	0.470	0.437	0.910	0.455
	GPSFun	0.477	0.552	0.948	0.512	0.486	**0.926**	**0.535**
RNA	aaRNA	0.422	0.360	0.870	0.389	0.318	0.803	0.359
	GeoBind	0.562	0.455	0.891	0.503	0.446	0.804	0.459
	GraphBind	0.633	0.400	0.871	0.491	0.436	0.861	0.506
	GPSFun	0.552	0.552	0.912	0.552	0.504	**0.901**	**0.578**
Peptide	PepBind	0.062	0.576	0.956	0.112	0.178	0.655	0.148
	PepNN	0.337	0.210	0.913	0.259	0.222	0.783	0.187
	PepBCL	0.168	0.389	0.951	0.234	0.233	0.758	0.222
	GPSFun	0.195	0.591	0.958	0.294	0.324	**0.846**	**0.344**
Protein	MaSIF-site	0.584	0.330	0.767	0.421	0.308	0.777	0.384
	GraphPPIS	0.670	0.320	0.745	0.434	0.328	0.794	0.422
	ScanNet	0.568	0.442	0.832	0.497	0.403	0.832	0.476
	GPSFun	0.613	0.419	0.820	0.498	0.403	**0.834**	**0.485**
ATP	GraphBind	0.529	0.473	0.967	0.499	0.483	0.901	0.503
	GeoBind	0.614	0.479	0.967	0.538	0.526	0.927	0.534
	DELIA	0.453	0.689	0.977	0.547	0.548	0.918	0.559
	GPSFun	0.720	0.678	0.981	0.698	0.688	**0.978**	**0.723**
HEM	GraphBind	0.733	0.505	0.939	0.598	0.578	0.926	0.638
	DELIA	0.604	0.670	0.957	0.636	0.614	0.928	0.664
	GeoBind	0.707	0.710	0.964	0.709	0.689	0.932	0.724
	GPSFun	0.707	0.787	0.970	0.745	0.730	**0.973**	**0.811**
Zn^2+^	TargetS	0.454	0.749	0.987	0.566	0.578	0.874	0.593
	IonCom	0.852	0.137	0.898	0.236	0.317	0.937	0.671
	LMetalSite	0.681	0.859	0.992	0.760	0.761	0.976	0.803
	GPSFun	0.710	0.910	0.993	0.798	0.801	**0.982**	**0.858**
Ca^2+^	GeoBind	0.279	0.515	0.985	0.362	0.372	0.895	0.348
	GraphBind	0.371	0.623	0.987	0.465	0.475	0.888	0.430
	LMetalSite	0.413	0.724	0.988	0.526	0.542	0.905	0.492
	GPSFun	0.398	0.848	0.990	0.542	0.577	**0.927**	**0.578**
Mg^2+^	GeoBind	0.181	0.475	0.990	0.263	0.289	0.840	0.227
	GraphBind	0.273	0.414	0.989	0.329	0.331	0.776	0.231
	LMetalSite	0.245	0.728	0.991	0.367	0.419	0.865	0.316
	GPSFun	0.263	0.732	0.992	0.387	0.436	**0.895**	**0.369**
Mn^2+^	GeoBind	0.569	0.479	0.988	0.520	0.516	0.938	0.454
	GraphBind	0.427	0.706	0.992	0.532	0.545	0.930	0.555
	LMetalSite	0.613	0.719	0.993	0.662	0.661	0.966	0.625
	GPSFun	0.662	0.730	0.994	0.695	0.692	**0.981**	**0.719**

*Note*: The best/second-best AUC and AUPR values are indicated by bold/underlined fonts.

For GO predictions, GPSFun achieves superior performance to sequence-based methods BLAST-KNN, DeepGOPlus (10) and GOLabeler (50), predicted structure-based method Foldseek-KNN, as well as protein-protein interaction network-based methods DeepGraphGO (11) and NetGO (13), by more than 11.6%, 25.3% and 5.8% in AUPR on the test sets of MF, BP and CC, respectively (Supplementary Table S7). Besides, GPSFun performs comparably to our previous SPROF-GO tool (12). GPSFun also generalizes well to non-homologous proteins as shown in Supplementary Table S8. Regarding subcellular localization prediction, GPSFun outperforms sequence-based predictors DeepLoc (16) and DeepLoc 2.0 (17) by more than 8.7% and 10.7% in micro and macro AUPR, respectively (see Table 2, Supplementary Table S9 and Supplementary Figure S3). GPSFun also exhibits improved performance compared to BLAST-KNN, Foldseek-KNN and the baseline model without structure information (Supplementary Table S10). For solubility prediction, GPSFun surpasses sequence-based predictors including GraphSol (18), SoluProt (19), SWI (51) and NetSolP (20) by more than 4.2% in AUC and 2.9% in AUPR (see Table 3 and Supplementary Figure S4). Similarly, GPSFun also outperforms BLAST-KNN, Foldseek-KNN and the baseline model without structures (Supplementary Table S11). Supplementary Tables S12–S15 also attest to the robustness of GPSFun according to the standard deviations of the models, as well as the benefits of the model ensemble technique.

**Table 2.** Performance comparison of GPSFun with state-of-the-art methods on the subcellular localization test set

	Micro			Macro				
Method	AUC	AUPR	F1	AUC	AUPR	F1	Acc	Jaccard
DeepLoc	0.812	0.599	0.487	0.726	0.458	0.368	0.360	0.404
DeepLoc 2.0	0.840	0.644	0.595	0.776	0.486	0.425	0.391	0.522
GPSFun	**0.876**	**0.700**	**0.629**	**0.802**	**0.538**	**0.483**	**0.416**	**0.551**

*Note*: Bold and underlined fonts indicate the best and second-best results, respectively.

**Table 3.** Performance comparison of GPSFun with state-of-the-art methods on the solubility test set

Method	Acc	MCC	AUC	AUPR
GraphSol	0.628	0.181	0.606	0.723
SoluProt	0.624	0.187	0.634	0.748
SWI	0.680	0.269	0.690	0.784
NetSolP	0.728	0.402	0.760	0.835
GPSFun	**0.734**	**0.435**	**0.792**	**0.859**

*Note*: Bold and underlined fonts indicate the best and second-best results, respectively.

## Conclusions

Despite the availability of numerous protein function predictors tailored for diverse tasks, there is still a lack of a convenient computational platform for high-quality predictions that comprehensively cover a broad range of functions. Moreover, most existing sequence-based predictors are computationally intensive due to their reliance on MSA, and limited in accuracy owing to the absence of structural information. On the other hand, existing experimental structure-based approaches are hampered in genome-scale applications for novel proteins with unsolved structures.

Building upon our prior well-validated methods for protein function annotations at residue and protein levels, we present GPSFun, a versatile web server designed to annotate various functions for protein sequences, including protein–ligand binding sites, gene ontologies, subcellular locations and solubility. GPSFun is equipped with sequence embeddings and predicted structures from large language models, along with an advanced geometric protein encoder. Consequently, GPSFun achieves superior performance to state-of-the-art methods, while eliminating the need for MSA and experimental protein structures. The user-friendly interfaces and rich interactive visualizations offered by GPSFun enable biologists and chemists without programming backgrounds to readily understand the results. Serving as a reliable and efficient tool, GPSFun could facilitate the exploration of the intricate landscape of protein functions, thereby bridging the gap between genome and phenome.

## Supplementary Material

gkae381_Supplemental_File

## Data Availability

The source code of GPSFun and the data underlying this article are available in figshare, at https://doi.org/10.6084/m9.figshare.25324903.
