# Supplementary material for: GPSFun: geometry-aware protein sequence function predictions with language models
Source: Nucleic Acids Res. 2024 May 13;52(W1):W248–55. doi: 10.1093/nar/gkae381 (PMC11223820; doi:10.1093/nar/gkae381)
Supplement: gkae381_Supplemental_File [file gkae381_supplemental_file.pdf]

Supplementary Materials for

GPSFun: geometry-aware protein sequence function predictions with language models

Qianmu Yuan[1], Chong Tian[1], Yidong Song[1], Peihua Ou[1], Mingming Zhu[1], Huiying Zhao[2,*] and Yuedong Yang[1,*]

[1] School of Computer Science and Engineering, Sun Yat-sen University, Guangzhou, Guangdong, 510000, China

[2] Sun Yat-sen Memorial Hospital, Sun Yat-sen University, Guangzhou, Guangdong, 510000, China

[*]To whom correspondence should be addressed. Tel: +86 020-37106046; E-mail: yangyd25@mail.sysu.edu.cn

Correspondence may also be addressed to Huiying Zhao. Email: zhaohy8@mail.sysu.edu.cn

The Supplementary Materials include:

Notes S1 to S4;

Tables S1 to S15;

Figures S1 to S4.

Note S1. Details of the benchmark datasets

The benchmark datasets for assessing binding site predictions of DNA, RNA, peptide, ATP, and HEM are compiled from BioLiP as discussed in Material and Methods. BioLiP is a database of biologically relevant protein-ligand complexes primarily from PDB, in which a binding residue is defined if the smallest atomic distance between the target residue and the ligand is < 0.5 Å plus the sum of the Van der Waal's radius of the two nearest atoms. Besides, the benchmark dataset of protein-protein binding sites is directly from (1), which contains non-redundant (pairwise sequence identity < 25%) transient heterodimeric protein complexes dated up to May 2021 collected from PDB. A protein-protein binding residue is defined as a surface residue (relative solvent accessibility > 5%) that lost more than 1 $Å^2$ absolute solvent accessibility after protein-protein complex formation. The benchmark datasets of metal ion (Zn^{2+}, Ca^{2+}, Mg^{2+} and Mn^{2+}) binding sites are directly from (2), which contain non-redundant (pairwise sequence identity < 25%) proteins dated up to 29 December 2021 from BioLiP. Proteins released before 1 January 2020 are used for training, while those released thereafter are used for testing. Combining all these ten datasets results in a total of 8441 training sequences and 1838 test sequences. Details of the statistics of these binding site benchmark datasets are given in Table S1.

To evaluate GO function predictions, we adopted the benchmark datasets proposed in (3), in which the training and test sets were collected following the standard protocol of the critical assessment of functional annotation (CAFA) competitions (4-6). Specifically, the GO term annotations were extracted and combined from Swiss-Prot (7), GOA (8) and GO (9) in January 2020. Only experimental annotations with the following evidence codes were kept: IDA, IPI, EXP, IGI, IMP, IEP, IC or TA. The annotations were further up-propagated based on the "is-a" relationship in the hierarchical structure of GO, and the root GO terms were omitted. Then, the training, validation and test sets were split according to the annotation time stamps. The training sets contain proteins annotated before January 2018, while the validation and test sets contain no-knowledge proteins annotated from January to December 2018 and from January 2019 to January 2020, respectively. In the training steps, only the GO terms with enough training samples (≥ 50 sequences) were considered, resulting in 790, 4766 and 667 classes for the molecular function (MF), biological process (BP), and cellular component (CC) sub-ontology. In the evaluation phase, we considered all terms to ensure fair comparisons with other methods. Table S2 shows the detailed statistics of the datasets for GO.

To evaluate subcellular localization prediction, we adopted the benchmark datasets from (10). The training set was originally extracted from UniProt (11) (release 2021_03), where the localization annotations were filtered using the following criteria: eukaryotic, not fragments, encoded in the nucleus, > 40 amino acids, and experimentally annotated (ECO:0000269) subcellular localizations. The proteins can be categorized into one or multiple of the ten locations: Cytoplasm, Nucleus, Cell membrane, Extracellular, Mitochondrion, Endoplasmic reticulum, Lysosome/Vacuole, Golgi apparatus, Plastid, and Peroxisome. The test set was derived from the Human Protein Atlas (HPA) project (12), in which only annotations with reliability labels of "Enhanced" and "Supported" were kept. Redundant sequences sharing identity > 30% with the training set were removed. Tables S3 and S4 present the statistics of these two subcellular localization datasets.

To evaluate protein solubility prediction, we adopted the benchmark datasets in (13). The training set was derived from a subset of the TargetTrack database by the Protein Structure Initiative (PSI) (14), in which the percentage of soluble proteins is ~66%. The North East Structural Consortium expressed proteins in *E. coli* using a unified production pipeline and provided integer scores (0-5) for both expression (E) and solubility (S) (15). The highly expressed proteins (E = 4 or 5) with high solubility scores (S = 4 or 5) were defined as the soluble proteins for the test set while proteins with low solubility scores (S = 0) were defined as insoluble. Using this definition, soluble proteins are ~64% of the test set. The test set was ensured not to have any sequences sharing sequence identity > 25% with the training set. Table S3 presents the statistics of these two solubility datasets.

Note S2. The geometric featurizer

GPSFun represents the protein as a radius graph derived from the C_α coordinates of the residues, where the radius is equal to 15 Å. An end-to-end featurizer is utilized for geometric feature extraction similar to (16), except that we additionally encode the sidechain conformations of the residues. Specifically, a local coordinate system is first defined at each residue based on the relative position of the C_α atom to other backbone atoms. Then, several geometric node and edge features are derived to capture the arrangements of backbone and sidechain atoms in or between residues.

(i) Local coordinate system. We define a local coordinate system $\boldsymbol{Q}_i = [\boldsymbol{b}_i, \boldsymbol{n}_i, \boldsymbol{b}_i \times \boldsymbol{n}_i]$ for residue i, where $\boldsymbol{b}_i$ is the negative bisector of the angle formed by the N, C_α, and C atoms, and $\boldsymbol{n}_i$ is a unit vector normal to this plane. Formally, we have:

$$\boldsymbol{u}_i = C_{\alpha_i} - N_i, \quad \boldsymbol{v}_i = C_i - C_{\alpha_i}, \quad \boldsymbol{b}_i = \frac{\boldsymbol{u}_i - \boldsymbol{v}_i}{\|\boldsymbol{u}_i - \boldsymbol{v}_i\|}, \quad \boldsymbol{n}_i = \frac{\boldsymbol{u}_i \times \boldsymbol{v}_i}{\|\boldsymbol{u}_i \times \boldsymbol{v}_i\|} \tag{1}$$

Based on the local coordinate systems, we could construct geometric features that are invariant to rotation and translation for single or pair of residues.

(ii) Geometric node features. GPSFun constructs distance, direction and angle features for each residue. Given the coordinates of two atoms A and B, the distance feature is computed via $\mathrm{RBF}(\|A - B\|)$, where $\mathrm{RBF}(\cdot)$ is a radial basis function. For the intra-residue distance features of node i, $A, B \in \{N_i, C_{\alpha_i}, C_i, O_i, R_i\}$ and $A \neq B$. Here, R denotes the centroid of the heavy sidechain atoms. The direction features encoding relative directions of other inner atoms to C_α in residue i are computed via $\boldsymbol{Q}_i^T \frac{A - C_{\alpha_i}}{\|A - C_{\alpha_i}\|}$, where $A \in \{N_i, C_i, O_i, R_i\}$. We also incorporate the sine and cosine values of the bond angles $(\alpha_i, \beta_i, \gamma_i)$ and torsion angles $(\phi_i, \psi_i, \omega_i)$ to consider the backbone geometry.

(iii) Geometric edge features. Similarly, we construct geometric features between neighboring residues including distance, direction and angle. The inter-residue distance features $\mathrm{RBF}(\|A - B\|)$ between nodes i and j are computed with atoms $A \in \{N_i, C_{\alpha_i}, C_i, O_i, R_i\}$ and $B \in \{N_j, C_{\alpha_j}, C_j, O_j, R_j\}$. The edge direction features $\boldsymbol{Q}_i^T \frac{A - C_{\alpha_i}}{\|A - C_{\alpha_i}\|}$ consider relative directions of all atoms in residue j to C_{α_i}, namely $A \in \{N_j, C_{\alpha_j}, C_j, O_j, R_j\}$. To reflect the relative spatial rotation between the two reference frames of residues i and j, the angle features $\mathrm{q}(\boldsymbol{Q}_i^T \boldsymbol{Q}_j)$ are employed, where $\mathrm{q}(\cdot)$ is the quaternion encoding function representing 3D rotation matrices as four-element vectors (17).

Note S3. Implementations of the baseline methods

Here we only discuss and compare GPSFun with the latest state-of-the-art methods in our benchmark experiments. For protein-ligand binding site predictions, we did not consider methods using PROSITE patterns (18) due to their historically demonstrated low coverage and sensitivity, as evidenced by previous studies (19,20). We also did not consider methods solely based on simple physicochemical features (e.g., AAindex (21)) since they have already been widely included in many competing methods such as GraphBind (22) and aaRNA (23). Specifically, we compared GPSFun with state-of-the-art sequence-based methods including GraphSite (24), PepBind (25), PepBCL (26), TargetS (27), and LMetalSite (2), as well as experimental structure-based methods including GraphBind (22), GeoBind (28), aaRNA (23), PepNN (29), MaSIF-site (30), GraphPPIS (1), ScanNet (31), DELIA (32), and IonCom (33). The implementations of these baseline methods are detailed as follows:

GraphSite: We used its standalone program with pre-trained model weights from https://github.com/biomed-

AI/GraphSite for inference and evaluation.

PepBind: We used its webserver (http://yanglab.nankai.edu.cn/PepBind/) for evaluation.

PepBCL: We adopted its standalone code with pre-trained model weights for inference and evaluation (https://github.com/Ruheng-W/PepBCL). There are two PepBCL models trained on two different datasets (1154 vs 640 training proteins), and here we report the results of the model trained on the larger dataset, since it performs slightly better.

TargetS: The results of TargetS were directly obtained from our previous study (2), which were originally obtained by running its webserver (http://www.csbio.sjtu.edu.cn/TargetS/) for evaluation.

LMetalSite: The results of LMetalSite were directly obtained from our previous study (2).

GraphBind: We used its standalone program with pre-trained model weights from http://www.csbio.sjtu.edu.cn/bioinf/GraphBind/sourcecode.html for inference and evaluation.

GeoBind: We used its webserver (http://www.zpliulab.cn/GeoBind/) for evaluation.

aaRNA: We used its webserver (https://sysimm.ifrec.osaka-u.ac.jp/aarna/) for evaluation.

PepNN: We used its standalone program with pre-trained model weights from https://gitlab.com/oabdin/pepnn for inference and evaluation.

MaSIF-site: The predictions of MaSIF-site for the test proteins were directly obtained from our previous work (1), which were originally generated by the standalone program with pre-trained model weights through a docker container from https://github.com/lpdi-epfl/masif.

GraphPPIS: The results of GraphPPIS were directly obtained from our previous study (1).

ScanNet: We used its standalone program with pre-trained model weights from https://github.com/jertubiana/ScanNet for inference and evaluation.

DELIA: We used its webserver (http://www.csbio.sjtu.edu.cn/bioinf/delia/) for evaluation.

IonCom: The results of IonCom were directly obtained from our previous study (2), which were originally obtained using its standalone program with pre-trained weights from https://zhanggroup.org/IonCom/.

In addition, we also implemented a simple baseline using a transformer (34) model solely fed with the PSSM and HMM evolutionary profiles from MSA (1,24). This model was trained separately for different ligands (i.e., in a single-task fashion), and the results are shown in Table S6.

For GO predictions, we compared GPSFun with state-of-the-art sequence-based methods DeepGOPlus (35), GOLabeler (36) and SPROF-GO (37), as well as protein-protein interaction network-based methods DeepGraphGO (3) and NetGO (38). In addition, two simple baseline methods named BLAST-KNN and Foldseek-KNN were also implemented as follows:

The idea of BLAST-KNN is that similar proteins may have similar functions. For a test protein p_i, BLAST (39) is run with an e-value cut-off of 0.001 to search p_i against all proteins in the training set to obtain its homologous protein set H_i. Then the probability score between p_i and a GO term G_j is computed as follows:

$$S(p_i, G_j) = \frac{\sum_{p \in H_i} I(p, G_j) \times B(p_i, p)}{\sum_{p \in H_i} B(p_i, p)} \quad (2)$$

where $B(p_i, p)$ is the bit-score between p_i and p, and $I(p, G_j)$ is a binary indicator which equals to 1 if protein p has the function G_j, or equals to 0 otherwise. The results of all these methods were directly obtained from previous works (3,37). Foldseek-KNN is similar to BLAST-KNN, except that Foldseek (40) is run to search the ESMFold-predicted structure of p_i against all protein structures in the training set, and TM-score is adopted as similarity measurement instead of bit-score.

For subcellular localization prediction, we compared GPSFun with state-of-the-art sequence-based predictors

DeepLoc (41) and DeepLoc 2.0 (10). Their results were obtained using their webservers (https://services.healthtech.dtu.dk/services/DeepLoc-1.0/ and https://services.healthtech.dtu.dk/services/DeepLoc-2.0/, respectively). Note that our evaluation results closely align with the previously reported results in (10). We also implemented two simple baselines, BLAST-KNN and Foldseek-KNN, as in the GO prediction tasks.

For protein solubility prediction, we compared GPSFun with state-of-the-art sequence-based predictors GraphSol (42), SoluProt (43), SWI (14), and NetSolP (13). The implementations of these baseline methods are detailed as follows:

GraphSol: We used its standalone program with pre-trained model weights from https://github.com/jcchan23/GraphSol for inference and evaluation.

SoluProt: We used its standalone program with pre-trained model weights from https://loschmidt.chemi.muni.cz/soluprot/?page=download for inference and evaluation.

SWI: We used its webserver (https://tisigner.com/sodope) for evaluation.

NetSolP: We used its standalone program with pre-trained model weights from https://github.com/tvinet/NetSolP-1.0 for inference and evaluation.

Note that the results of SoluProt, SWI and NetSolP that we obtained are consistent with those reported in (13). We also implemented two simple baselines, BLAST-KNN and Foldseek-KNN, as in the GO prediction tasks.

Note S4. Evaluation metrics

Following the previous studies, we employ recall (Rec), precision (Pre), accuracy (Acc), F1-score (F1), Matthews correlation coefficient (MCC), area under the receiver operating characteristic curve (AUC), and area under the precision-recall curve (AUPR) to evaluate the performance of protein-ligand binding site predictions:

$$Rec = \frac{TP}{TP + FN} \tag{3}$$

$$Pre = \frac{TP}{TP + FP} \tag{4}$$

$$Acc = \frac{TP + TN}{TP + TN + FP + FN} \tag{5}$$

$$F1 = 2 \times \frac{Pre \times Rec}{Pre + Rec} \tag{6}$$

$$MCC = \frac{TP \times TN - FN \times FP}{\sqrt{(TP + FP) \times (TP + FN) \times (TN + FP) \times (TN + FN)}} \tag{7}$$

where true positives (TP) and true negatives (TN) denote the numbers of correctly predicted binding and non-binding residues, and false positives (FP) and false negatives (FN) denote the numbers of incorrectly predicted binding and non-binding residues, respectively. AUC and AUPR are independent of thresholds, thus reflecting the overall performance of a model. The other metrics are calculated using a threshold to convert the predicted binding probabilities to binary predictions. We go through 101 thresholds from 0 to 1 with an interval of 0.01, and select the best threshold that maximizes the MCC on the validation sets.

We assess the predictive performance for the three domains in GO (MF, BP, and CC) independently using maximum protein-centric F-measure (F_{max}) and AUPR. F_{max} is computed as follows:

$$F_{max} = \max_{\tau}\left\{2 \cdot \frac{\overline{pr}(\tau) \cdot \overline{rc}(\tau)}{\overline{pr}(\tau) + \overline{rc}(\tau)}\right\} \tag{8}$$

where $\overline{pr}(\tau)$ and $\overline{rc}(\tau)$ denote the average precision and recall at threshold τ respectively, which are defined as follows:

$$\overline{pr}(\tau) = \frac{1}{M(\tau)} \sum_{i=1}^{M(\tau)} \frac{|T_i \cap P_i(\tau)|}{|P_i(\tau)|} \tag{9}$$

$$\overline{rc}(\tau) = \frac{1}{N_T} \sum_{i=1}^{N_T} \frac{|T_i \cap P_i(\tau)|}{|T_i|} \tag{10}$$

where $M(\tau)$ denotes the number of proteins predicted with at least one GO term at threshold τ; N_T denotes the total number of proteins; T_i denotes the true annotation set for protein i; $P_i(\tau)$ denotes the predicted annotation set for protein i at threshold τ; and $|\cdot|$ is the set cardinality operation. We use 101 thresholds from 0 to 1 with an interval of 0.01 to compute the above measurements. The AUPR score is calculated from the computed precision and recall scores using the trapezoidal rule.

To assess the performance of subcellular localization prediction, we use accuracy, Jaccard, micro/macro F1, micro/macro AUC, and micro/macro AUPR. Here, the calculation of accuracy requires the exact location(s) to be predicted. Jaccard measures the intersection of the actual and predicted labels over their union. Micro metrics are calculated by aggregating the labels from all classes and then computing the metrics globally, whereas macro metrics are calculated by computing the metrics for each class and then averaging them.

To evaluate the performance of solubility prediction, we use accuracy, MCC and AUC following the previous works. The threshold used to convert the predicted solubility probabilities to binary predictions was selected by maximizing the MCC on the validation sets.

Table S1. Statistics of the ten binding site benchmark datasets

Molecule type	Train			Test		
	Sequences	Residues	% of binding residues	Sequences	Residues	% of binding residues
DNA	661	185,796	8.06	146	57,914	5.75
RNA	689	205,648	10.55	346	105,230	9.78
Peptide	1251	348,370	5.39	235	74,788	4.50
Protein	335	66,366	15.63	375	78,475	14.57
ATP	347	130,655	3.91	79	39,459	3.12
HEM	176	47,063	8.55	48	15,618	6.21
Zn^{2+}	1646	474,855	1.63	211	56,020	1.85
Ca^{2+}	1554	504,146	1.67	183	66,854	1.55
Mg^{2+}	1729	575,732	1.10	235	88,806	1.01
Mn^{2+}	547	181,699	1.41	57	20,419	1.10

Note: We combined the two test sets (Test_60 and Test_315) from (1) to establish our final protein-protein binding site test set.

Table S2. Numbers of proteins in the training, validation and test sets for the three domains in GO, i.e., molecular function (MF), biological process (BP), and cellular component (CC)

Domain	Train	Valid	Test
MF	51,549	490	426
BP	85,104	1570	925
CC	76,098	923	1224

Table S3. Numbers of proteins in the training and test sets for subcellular localization and solubility predictions

Task	Train	Test
Subcellular localization	28,304	1717
Solubility	11,226	1323

Table S4. Numbers of proteins in each location in the training and test sets for subcellular localization prediction

Location	Train	Test
Cytoplasm	9870	562
Nucleus	9720	893
Cell membrane	4187	287
Extracellular	3301	0
Mitochondrion	2590	196
Endoplasmic reticulum	2180	77
Lysosome/Vacuole	1496	2
Golgi apparatus	1279	86
Plastid	1047	0
Peroxisome	304	7

Table S5. The details of the architectures and training strategies of the models in GPSFun

Task	Protein binding site	Gene Ontology			Subcellular localization	Solubility
		Molecular Function	Biological Process	Cellular Component		
Output size	10	790	4766	667	10	1
Parameters	1,668,490	8,896,222	15,792,998	7,394,147	473,866	3,130,197
GNN layers	4	2	1	1	4	2
Hidden units	128	256	256	256	64	256
Dropout	0.2	0.2	0.2	0.2	0.2	0.2
Learning rate	0.001	0.001	0.0001	0.0001	0.0002	0.001
Training epochs	75	30	30	30	50	50
Patience of early stopping	10	4	4	4	10	10
Batch size	16	20	20	20	16	16

Table S6. The ablation studies on protein features and model designs in the ten binding site test sets

Method	DNA	RNA	Pep	Pro	ATP	HEM	Zn^{2+}	Ca^{2+}	Mg^{2+}	Mn^{2+}	Avg
Baseline	0.228	0.366	0.104	0.249	0.280	0.309	0.684	0.208	0.135	0.394	0.296
MSA profiles	0.509	0.562	0.304	0.473	0.683	0.809	0.822	0.540	0.364	0.693	0.576
w/o structure	0.432	0.503	0.245	0.395	0.537	0.569	0.792	0.467	0.285	0.612	0.484
w/o geometry	0.485	0.542	0.304	0.443	0.621	0.672	0.806	0.483	0.315	0.644	0.531
GPSFun	**0.535**	**0.578**	**0.344**	**0.485**	**0.723**	**0.811**	**0.858**	**0.578**	**0.369**	**0.719**	**0.600**

Note: The numbers in this table are AUPR values. Bold fonts indicate the best results. "Pep" and "Pro" denote peptide and protein, respectively. "Avg" means the average AUPR values among the ten test sets. "Baseline" means using a transformer (34) model fed with the PSSM and HMM evolutionary profiles from MSA (1,24). This model was trained separately for different ligands (i.e., in a single-task fashion). "MSA profiles" means replacing the ProtTrans embeddings with PSSM and HMM. "w/o structure" means using a transformer model fed with the ProtTrans sequence features. "w/o geometry" means removing the geometric featurizer in GPSFun.

Table S7. Performance comparison of GPSFun with state-of-the-art methods on the test sets of the three domains in GO

Method	F_{max}			AUPR		
	MF	BP	CC	MF	BP	CC
BLAST-KNN	0.590	0.274	0.650	0.455	0.113	0.570
Foldseek-KNN	0.544	0.277	0.660	0.508	0.175	0.680
DeepGOPlus	0.593	0.290	0.672	0.398	0.108	0.595
GOLabeler	0.629	0.296	0.685	0.558	0.149	0.708
DeepGraphGO	0.623	0.327	0.692	0.543	0.194	0.695
NetGO	0.630	0.335	0.697	0.553	0.190	0.725
SPROF-GO	**0.647**	0.335	0.725	0.622	**0.247**	0.765
GPSFun	0.641	**0.336**	**0.726**	**0.623**	0.243	**0.767**

Note: See Note S3 for the implementation details of BLAST-KNN and Foldseek-KNN. Bold and underlined fonts indicate the best and second-best results, respectively.

Table S8. Performance comparison of GPSFun with state-of-the-art methods on *difficult* proteins within the test sets of the three domains in GO

Method	F_{max}			AUPR		
	MF	BP	CC	MF	BP	CC
BLAST-KNN	0.534	0.274	0.521	0.377	0.114	0.354
Foldseek-KNN	0.528	0.270	0.609	0.486	0.172	0.596
DeepGOPlus	0.564	0.292	0.602	0.326	0.108	0.454
DeepGraphGO	0.598	0.322	0.625	0.508	0.184	0.607
SPROF-GO	**0.630**	0.339	0.682	**0.617**	**0.256**	0.708
GPSFun	0.623	**0.340**	**0.683**	0.610	0.251	**0.710**

Note: The *difficult* proteins are defined by CAFA2 (5) as the test proteins with sequence identity <60% to the training set. The numbers of *difficult* proteins in the MF, BP and CC test sets are 303, 649 and 437, respectively. See Note S3 for the implementation details of BLAST-KNN and Foldseek-KNN. Bold and underlined fonts indicate the best and second-best results, respectively.

Table S9. Performance comparison of GPSFun with state-of-the-art methods in each location on the subcellular localization test set

Location		Cytoplasm	Nucleus	Cell membrane	Mitochondrion	Endoplasmic reticulum	Golgi apparatus
AUC	DeepLoc	0.705	0.772	0.650	0.861	0.649	0.717
	DeepLoc 2.0	0.746	0.804	0.770	0.879	**0.736**	0.722
	GPSFun	**0.755**	**0.832**	**0.827**	**0.912**	0.725	**0.763**
AUPR	DeepLoc	0.516	0.790	0.358	0.660	**0.178**	0.248
	DeepLoc 2.0	0.556	0.838	0.489	0.651	0.143	0.238
	GPSFun	**0.564**	**0.857**	**0.553**	**0.748**	0.166	**0.339**

Note: The locations with less than 10 test proteins are omitted. Bold and underlined fonts indicate the best and second-best results, respectively.

Table S10. Performance comparison of GPSFun with three baseline methods on the subcellular localization test set

Method	Micro			Macro			Acc	Jaccard
	AUC	AUPR	F1	AUC	AUPR	F1		
BLAST-KNN	0.817	0.609	0.477	0.693	0.456	0.377	0.274	0.339
Foldseek-KNN	0.838	0.625	0.493	0.701	0.420	0.342	0.303	0.368
GPSFun w/o structure	0.873	0.677	0.603	**0.810**	0.525	0.441	**0.418**	0.535
GPSFun	**0.876**	**0.700**	**0.629**	0.802	**0.538**	**0.483**	0.416	**0.551**

Note: See Note S3 for the implementation details of BLAST-KNN and Foldseek-KNN. "GPSFun w/o structure" means using a transformer model fed with the ProtTrans sequence features. Bold and underlined fonts indicate the best and second-best results, respectively.

Table S11. Performance comparison of GPSFun with three baseline methods on the solubility test set

Method	Acc	MCC	AUC	AUPR
BLAST-KNN	0.635	0.082	0.529	0.676
Foldseek-KNN	0.639	0.119	0.578	0.685
GPSFun w/o structure	**0.734**	0.402	0.777	0.844
GPSFun	**0.734**	**0.435**	**0.792**	**0.859**

Note: See Note S3 for the implementation details of BLAST-KNN and Foldseek-KNN. "GPSFun w/o structure" means using a transformer model fed with the ProtTrans sequence features. Bold and underlined fonts indicate the best and second-best results, respectively.

Table S12. The performance of GPSFun with or without model ensemble on the ligand-binding site test sets

Test set	Method	F1	MCC	AUC	AUPR
DNA	w/o ensemble	0.476±0.012	0.446±0.010	0.912±0.004	0.482±0.015
	GPSFun	0.512	0.486	0.926	0.535
RNA	w/o ensemble	0.528±0.004	0.476±0.004	0.888±0.004	0.539±0.006
	GPSFun	0.552	0.504	0.901	0.578
Peptide	w/o ensemble	0.299±0.027	0.295±0.016	0.818±0.008	0.290±0.013
	GPSFun	0.294	0.324	0.846	0.344
Protein	w/o ensemble	0.452±0.004	0.349±0.005	0.805±0.003	0.432±0.008
	GPSFun	0.498	0.403	0.834	0.485
ATP	w/o ensemble	0.654±0.006	0.643±0.006	0.967±0.004	0.675±0.008
	GPSFun	0.698	0.688	0.978	0.723
HEM	w/o ensemble	0.707±0.024	0.691±0.024	0.967±0.003	0.766±0.022
	GPSFun	0.745	0.730	0.973	0.811
Zn^{2+}	w/o ensemble	0.785±0.006	0.785±0.007	0.978±0.002	0.839±0.006
	GPSFun	0.798	0.801	0.982	0.858
Ca^{2+}	w/o ensemble	0.543±0.013	0.567±0.009	0.915±0.004	0.542±0.010
	GPSFun	0.542	0.577	0.927	0.578
Mg^{2+}	w/o ensemble	0.383±0.013	0.414±0.013	0.878±0.006	0.326±0.010
	GPSFun	0.387	0.436	0.895	0.369
Mn^{2+}	w/o ensemble	0.671±0.014	0.668±0.015	0.974±0.005	0.686±0.015
	GPSFun	0.695	0.692	0.981	0.719

Note: “w/o ensemble” reports the mean and standard deviation of the performance metrics of the five trained models from cross-validation.

Table S13. The performance of GPSFun with or without model ensemble on the GO test sets

Method	F_{max}			AUPR		
	MF	BP	CC	MF	BP	CC
w/o ensemble	0.630±0.010	0.332±0.004	0.719±0.003	0.606±0.010	0.239±0.002	0.755±0.003
GPSFun	0.641	0.336	0.726	0.623	0.243	0.767

Note: "w/o ensemble" reports the mean and standard deviation of the performance metrics of the five trained models from five different random seeds.

Table S14. The performance of GPSFun with or without model ensemble on the subcellular localization test set

Method	Micro			Macro			Acc	Jaccard
	AUC	AUPR	F1	AUC	AUPR	F1		
w/o ensemble	0.867 ±0.007	0.674 ±0.020	0.611 ±0.010	0.789 ±0.012	0.510 ±0.013	0.470 ±0.010	0.411 ±0.015	0.540 ±0.012
GPSFun	0.876	0.700	0.629	0.802	0.538	0.483	0.416	0.551

Note: "w/o ensemble" reports the mean and standard deviation of the performance metrics of the five trained models from cross-validation.

Table S15. The performance of GPSFun with or without model ensemble on the solubility test set

Method	Acc	MCC	AUC	AUPR
w/o ensemble	0.703±0.030	0.388±0.013	0.778±0.005	0.848±0.003
GPSFun	0.734	0.435	0.792	0.859

Note: "w/o ensemble" reports the mean and standard deviation of the performance metrics of the five trained models from cross-validation.

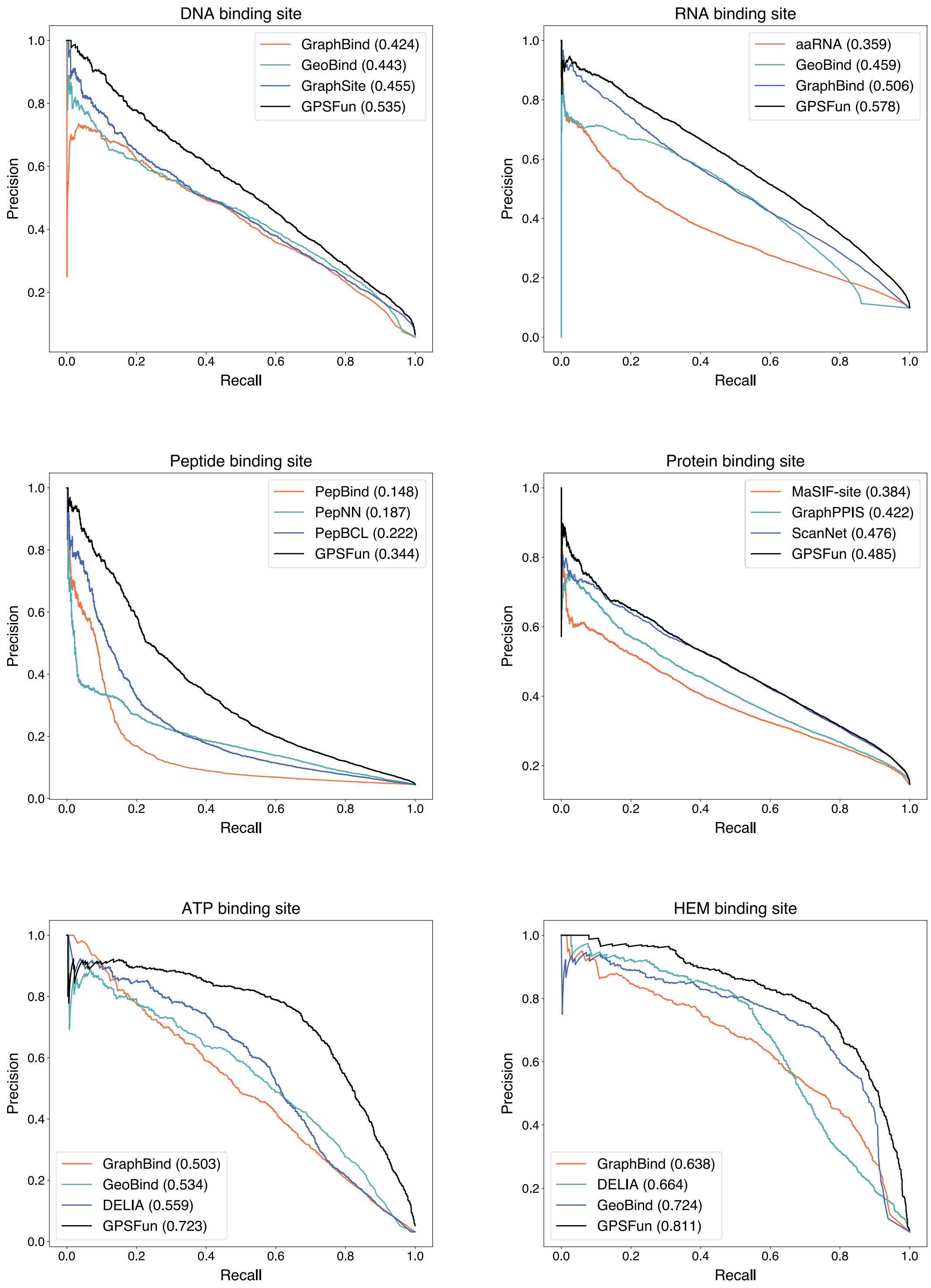

Figure S1. Precision-recall curves of GPSFun and other methods on the binding site test sets of DNA, RNA, peptide, protein, ATP and HEM.

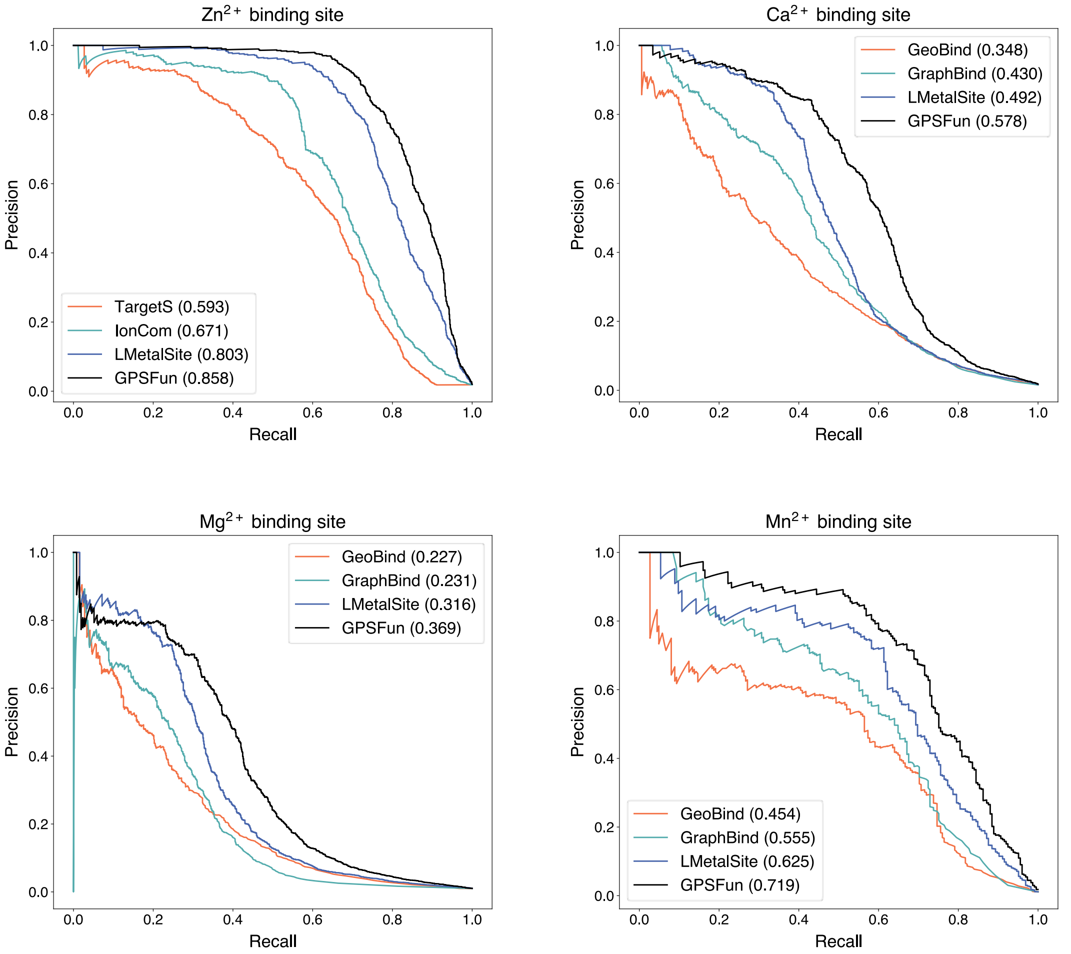

Figure S2. Precision-recall curves of GPSFun and other methods on the binding site test sets of Zn^{2+}, Ca^{2+}, Mg^{2+}, and Mn^{2+} ions.

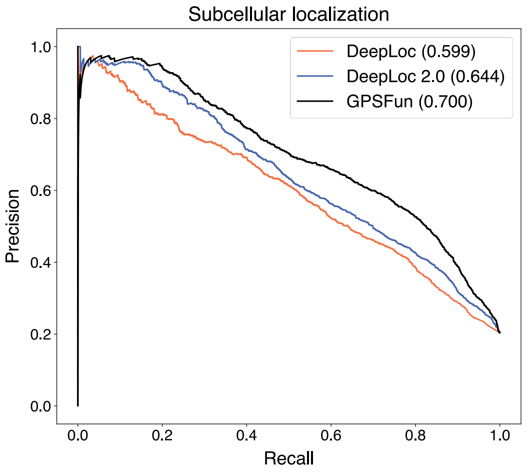

Figure S3. Precision-recall curves of GPSFun and other methods on the subcellular localization test set.

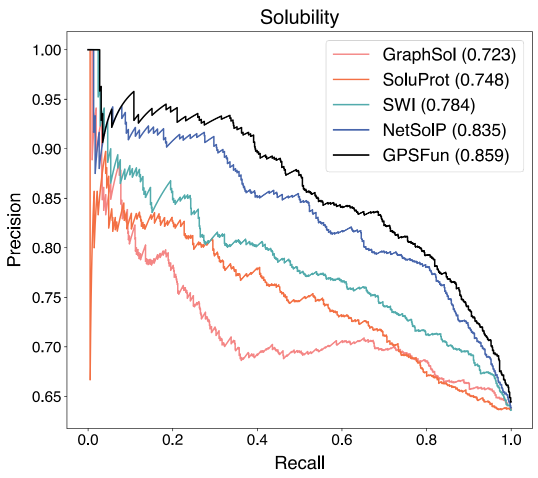

Figure S4. Precision-recall curves of GPSFun and other methods on the protein solubility test set.

Reference

1. Yuan, Q., Chen, J., Zhao, H., Zhou, Y. and Yang, Y. (2021) Structure-aware protein-protein interaction site prediction using deep graph convolutional network. *Bioinformatics (Oxford, England)*, **38**, 125-132.
2. Yuan, Q., Chen, S., Wang, Y., Zhao, H. and Yang, Y. (2022) Alignment-free metal ion-binding site prediction from protein sequence through pretrained language model and multi-task learning. *Briefings in bioinformatics*, **23**, ppublish.
3. You, R., Yao, S., Mamitsuka, H. and Zhu, S. (2021) DeepGraphGO: graph neural network for large-scale, multispecies protein function prediction. *Bioinformatics (Oxford, England)*, **37**, i262-i271.
4. Radivojac, P., Clark, W.T., Oron, T.R., Schnoes, A.M., Wittkop, T., Sokolov, A., Graim, K., Funk, C., Verspoor, K., Ben-Hur, A. *et al.* (2013) A large-scale evaluation of computational protein function prediction. *Nature methods*, **10**, 221-227.
5. Jiang, Y., Oron, T.R., Clark, W.T., Bankapur, A.R., D'Andrea, D., Lepore, R., Funk, C.S., Kahanda, I., Verspoor, K.M., Ben-Hur, A. *et al.* (2016) An expanded evaluation of protein function prediction methods shows an improvement in accuracy. *Genome biology*, **17**, 184.
6. Zhou, N., Jiang, Y., Bergquist, T.R., Lee, A.J., Kacsoh, B.Z., Crocker, A.W., Lewis, K.A., Georghiou, G., Nguyen, H.N., Hamid, M.N. *et al.* (2019) The CAFA challenge reports improved protein function prediction and new functional annotations for hundreds of genes through experimental screens. *Genome biology*, **20**, 244.
7. Boutet, E., Lieberherr, D., Tognolli, M., Schneider, M., Bansal, P., Bridge, A.J., Poux, S., Bougueleret, L. and Xenarios, I. (2016) UniProtKB/Swiss-Prot, the Manually Annotated Section of the UniProt KnowledgeBase: How to Use the Entry View. *Methods in molecular biology (Clifton, N.J.)*, **1374**, 23-54.
8. Huntley, R.P., Sawford, T., Mutowo-Meullenet, P., Shypitsyna, A., Bonilla, C., Martin, M.J. and O'Donovan, C. (2015) The GOA database: gene Ontology annotation updates for 2015. *Nucleic acids research*, **43**, D1057-1063.
9. Ashburner, M., Ball, C.A., Blake, J.A., Botstein, D., Butler, H., Cherry, J.M., Davis, A.P., Dolinski, K., Dwight, S.S., Eppig, J.T. *et al.* (2000) Gene ontology: tool for the unification of biology. The Gene Ontology Consortium. *Nature genetics*, **25**, 25-29.

10. Thumuluri, V., Almagro Armenteros, J.J., Johansen, A.R., Nielsen, H. and Winther, O. (2022) DeepLoc 2.0: multi-label subcellular localization prediction using protein language models. *Nucleic acids research*, **50**, W228-W234.

11. UniProt Consortium. (2023) UniProt: the Universal Protein Knowledgebase in 2023. *Nucleic acids research*, **51**, D523-D531.

12. Thul, P.J., Åkesson, L., Wiking, M., Mahdessian, D., Geladaki, A., Ait Blal, H., Alm, T., Asplund, A., Björk, L., Breckels, L.M. *et al.* (2017) A subcellular map of the human proteome. *Science (New York, N.Y.)*, **356**, ppublish.

13. Thumuluri, V., Martiny, H.M., Almagro Armenteros, J.J., Salomon, J., Nielsen, H. and Johansen, A.R. (2022) NetSolP: predicting protein solubility in Escherichia coli using language models. *Bioinformatics (Oxford, England)*, **38**, 941-946.

14. Bhandari, B.K., Gardner, P.P. and Lim, C.S. (2020) Solubility-Weighted Index: fast and accurate prediction of protein solubility. *Bioinformatics (Oxford, England)*, **36**, 4691-4698.

15. Price, W.N., 2nd, Handelman, S.K., Everett, J.K., Tong, S.N., Bracic, A., Luff, J.D., Naumov, V., Acton, T., Manor, P., Xiao, R. *et al.* (2011) Large-scale experimental studies show unexpected amino acid effects on protein expression and solubility in vivo in E. coli. *Microbial informatics and experimentation*, **1**, 6.

16. Gao, Z., Tan, C. and Li, S.Z. (2022) PiFold: Toward effective and efficient protein inverse folding. *arXiv preprint arXiv:2209.12643*.

17. Huynh, D.Q. (2009) Metrics for 3D rotations: Comparison and analysis. *Journal of Mathematical Imaging and Vision*, **35**, 155-164.

18. Hulo, N., Bairoch, A., Bulliard, V., Cerutti, L., De Castro, E., Langendijk-Genevaux, P.S., Pagni, M. and Sigrist, C.J. (2006) The PROSITE database. *Nucleic acids research*, **34**, D227-230.

19. Brylinski, M. and Skolnick, J. (2008) A threading-based method (FINDSITE) for ligand-binding site prediction and functional annotation. *Proceedings of the National Academy of Sciences of the United States of America*, **105**, 129-134.

20. Brylinski, M. and Skolnick, J. (2011) FINDSITE-metal: integrating evolutionary information and machine learning for structure-based metal-binding site prediction at the proteome level. *Proteins*, **79**, 735-751.

21. Kawashima, S. and Kanehisa, M. (2000) AAindex: amino acid index database. *Nucleic acids research*, **28**, 374.

22. Xia, Y., Xia, C.Q., Pan, X. and Shen, H.B. (2021) GraphBind: protein structural context embedded rules learned by hierarchical graph neural networks for recognizing nucleic-acid-binding residues. *Nucleic acids research*, **49**, e51.

23. Li, S., Yamashita, K., Amada, K.M. and Standley, D.M. (2014) Quantifying sequence and structural features of protein-RNA interactions. *Nucleic acids research*, **42**, 10086-10098.

24. Yuan, Q., Chen, S., Rao, J., Zheng, S., Zhao, H. and Yang, Y. (2022) AlphaFold2-aware protein-DNA binding site prediction using graph transformer. *Briefings in bioinformatics*, **23**, ppublish.

25. Zhao, Z., Peng, Z. and Yang, J. (2018) Improving Sequence-Based Prediction of Protein-Peptide Binding Residues by Introducing Intrinsic Disorder and a Consensus Method. *Journal of chemical information and modeling*, **58**, 1459-1468.

26. Wang, R., Jin, J., Zou, Q., Nakai, K. and Wei, L. (2022) Predicting protein-peptide binding residues via interpretable deep learning. *Bioinformatics (Oxford, England)*, **38**, 3351-3360.

27. Yu, D.J., Hu, J., Yang, J., Shen, H.B., Tang, J. and Yang, J.Y. (2013) Designing template-free predictor for targeting protein-ligand binding sites with classifier ensemble and spatial clustering. *IEEE/ACM transactions on computational biology and bioinformatics*, **10**, 994-1008.

28. Li, P. and Liu, Z.P. (2023) GeoBind: segmentation of nucleic acid binding interface on protein surface with geometric deep learning. *Nucleic acids research*, **51**, e60.
29. Abdin, O., Nim, S., Wen, H. and Kim, P.M. (2022) PepNN: a deep attention model for the identification of peptide binding sites. *Communications biology*, **5**, 503.
30. Gainza, P., Sverrisson, F., Monti, F., Rodolà, E., Boscaini, D., Bronstein, M.M. and Correia, B.E. (2020) Deciphering interaction fingerprints from protein molecular surfaces using geometric deep learning. *Nature methods*, **17**, 184-192.
31. Tubiana, J., Schneidman-Duhovny, D. and Wolfson, H.J. (2022) ScanNet: an interpretable geometric deep learning model for structure-based protein binding site prediction. *Nature methods*, **19**, 730-739.
32. Xia, C.Q., Pan, X. and Shen, H.B. (2020) Protein-ligand binding residue prediction enhancement through hybrid deep heterogeneous learning of sequence and structure data. *Bioinformatics (Oxford, England)*, **36**, 3018-3027.
33. Hu, X., Dong, Q., Yang, J. and Zhang, Y. (2016) Recognizing metal and acid radical ion-binding sites by integrating ab initio modeling with template-based transferals. *Bioinformatics (Oxford, England)*, **32**, 3260-3269.
34. Vaswani, A., Shazeer, N., Parmar, N., Uszkoreit, J., Jones, L., Gomez, A.N., Kaiser, Ł. and Polosukhin, I. (2017) Attention is all you need. *Advances in neural information processing systems*, **30**.
35. Kulmanov, M. and Hoehndorf, R. (2020) DeepGOPlus: improved protein function prediction from sequence. *Bioinformatics (Oxford, England)*, **36**, 422-429.
36. You, R., Zhang, Z., Xiong, Y., Sun, F., Mamitsuka, H. and Zhu, S. (2018) GOLabeler: improving sequence-based large-scale protein function prediction by learning to rank. *Bioinformatics (Oxford, England)*, **34**, 2465-2473.
37. Yuan, Q., Xie, J., Xie, J., Zhao, H. and Yang, Y. (2023) Fast and accurate protein function prediction from sequence through pretrained language model and homology-based label diffusion. *Briefings in bioinformatics*, **24**, ppublish.
38. You, R., Yao, S., Xiong, Y., Huang, X., Sun, F., Mamitsuka, H. and Zhu, S. (2019) NetGO: improving large-scale protein function prediction with massive network information. *Nucleic acids research*, **47**, W379-W387.
39. Altschul, S.F., Madden, T.L., Schäffer, A.A., Zhang, J., Zhang, Z., Miller, W. and Lipman, D.J. (1997) Gapped BLAST and PSI-BLAST: a new generation of protein database search programs. *Nucleic acids research*, **25**, 3389-3402.
40. van Kempen, M., Kim, S.S., Tumescheit, C., Mirdita, M., Lee, J., Gilchrist, C.L.M., Söding, J. and Steinegger, M. (2024) Fast and accurate protein structure search with Foldseek. *Nature biotechnology*, **42**, 243-246.
41. Almagro Armenteros, J.J., Sønderby, C.K., Sønderby, S.K., Nielsen, H. and Winther, O. (2017) DeepLoc: prediction of protein subcellular localization using deep learning. *Bioinformatics (Oxford, England)*, **33**, 3387-3395.
42. Chen, J., Zheng, S., Zhao, H. and Yang, Y. (2021) Structure-aware protein solubility prediction from sequence through graph convolutional network and predicted contact map. *Journal of cheminformatics*, **13**, 7.
43. Hon, J., Marusiak, M., Martinek, T., Kunka, A., Zendulka, J., Bednar, D. and Damborsky, J. (2021) SoluProt: prediction of soluble protein expression in Escherichia coli. *Bioinformatics (Oxford, England)*, **37**, 23-28.
